# Growth orientations, rather than heterogeneous growth rates, dominate jaw joint morphogenesis in the larval zebrafish

**DOI:** 10.1111/joa.13680

**Published:** 2022-05-05

**Authors:** Josepha Godivier, Elizabeth A. Lawrence, Mengdi Wang, Chrissy L. Hammond, Niamh C. Nowlan

**Affiliations:** ^1^ Imperial College London London United Kingdom; ^2^ University of Bristol Bristol UK; ^3^ University College Dublin Dublin Ireland

**Keywords:** computational simulation, finite element model, high‐resolution imaging, joint shape, skeletal development

## Abstract

In early limb embryogenesis, synovial joints acquire specific shapes which determine joint motion and function. The process by which the opposing cartilaginous joint surfaces are moulded into reciprocal and interlocking shapes, called joint morphogenesis, is one of the least understood aspects of joint formation and the cell‐level dynamics underlying it are yet to be unravelled. In this research, we quantified key cellular dynamics involved in growth and morphogenesis of the zebrafish jaw joint and synthesised them in a predictive computational simulation of joint development. Cells in larval zebrafish jaw joints labelled with cartilage markers were tracked over a 48‐h time window using confocal imaging. Changes in distance and angle between adjacent cell centroids resulting from cell rearrangement, volume expansion and extracellular matrix (ECM) deposition were measured and used to calculate the rate and direction of local tissue deformations. We observed spatially and temporally heterogeneous growth patterns with marked anisotropy over the developmental period assessed. There was notably elevated growth at the level of the retroarticular process of the Meckel's cartilage, a feature known to undergo pronounced shape changes during zebrafish development. Analysis of cell dynamics indicated a dominant role for cell volume expansion in growth, with minor influences from ECM volume increases and cell intercalation. Cell proliferation in the joint was minimal over the timeframe of interest. Synthesising the dynamic cell data into a finite element model of jaw joint development resulted in accurate shape predictions. Our biofidelic computational simulation demonstrated that zebrafish jaw joint growth can be reasonably approximated based on cell positional information over time, where cell positional information derives mainly from cell orientation and cell volume expansion. By modifying the input parameters of the simulation, we were able to assess the relative contributions of heterogeneous growth rates and of growth orientation. The use of uniform rather than heterogeneous growth rates only minorly impacted the shape predictions, whereas isotropic growth fields resulted in altered shape predictions. The simulation results suggest that growth anisotropy is the dominant influence on joint growth and morphogenesis. This study addresses the gap of the cellular processes underlying joint morphogenesis, with implications for understanding the aetiology of developmental joint disorders such as developmental dysplasia of the hip and arthrogryposis.

## Graphical abstract

Using a biofidelic computational simulation, we demonstrated that zebrafish jaw joint growth can be reasonably approximated based on cell positional information over time, where cell positional information derives mainly from cell orientation and volume expansion. Growth anisotropy was found to be the dominant influence on joint growth and morphogenesis, with growth heterogeneity only minorly impacting the shape predictions.

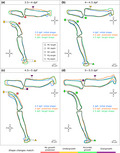

## 1 INTRODUCTION

Synovial joints are complex structures connecting skeletal elements while allowing different types of motion. In early limb embryogenesis, the cartilaginous anlagen of the future skeletal elements are initially uninterrupted (Yang, 2013). A zone of compact and interconnected cells called the interzone emerges, marking the location of the future joint. Physical separation of the skeletal elements occurs by cavitation of the interzone while the two opposing surfaces mould into reciprocal and interlocking shapes in a process known as joint morphogenesis (Chijimatsu & Saito, 2019; Pacifici et al., 2005; Rux et al., 2019). A variety of distinct and complex joint shapes, which are specific to anatomical sites and allow distinct motions, emerge from this process; examples of joint diversity are the hinge joint of the knee and the ball and socket of the hip. This process by which joints acquire their shapes has important ramifications for joint health and function. For example, sub‐optimal hip joint shape is believed to be a key risk factor in early‐onset osteoarthritis (Faber et al., 2020; Sandell, 2012). However, the mechanisms underlying the emergence of joint shape remain poorly understood.

A small number of studies have identified cell activities involved in joint growth and morphogenesis. Work on embryonic murine synovial joints has shown that a continuous influx of pro‐chondrogenic cells contributes to joint morphogenesis (Shwartz et al., 2016), with evidence that asymmetric influx and proliferation of these cells enable the emergence of asymmetric shape features (Zhang et al., 2020). Maintenance of cell fate has been shown to be essential for joint cavitation and morphogenesis. The absence of muscle contraction results in premature differentiation of joint pro‐chondrocytes with consequences for joint shape in the embryonic murine elbow (Kahn et al., 2009). The roles of cell size, orientation and intercalation in developing zebrafish and murine joints have been identified (Brunt et al., 2015; Shwartz et al., 2012) and differential cell volume expansion and cell rearrangements were shown to be key factors for thickening and organisation in postnatal murine articular cartilage (Decker et al., 2017). Cell proliferation and cell death do not majorly impact morphogenesis in postnatal murine articular cartilage (Decker et al., 2017). These observations provide insights on the cellular dynamics underlying joint morphogenesis, but there is a lack of understanding of the contribution of each of these processes to joint growth and morphogenesis. The research question we tackle in this paper is how a complex range of dynamic cellular activities combine to enable the formation of specific shape features in synovial joints.

Computational models enable the synthesis of experimental data and a means to test hypotheses via simulation. In previous work from our group, (Giorgi et al., 2014), the emergence of different joint shapes based on types of simulated fetal movements was predicted in a mechanobiological simulation. A simulation of hip joint development revealed how asymmetric movements can result in altered shapes resembling those seen in developmental dysplasia of the hip (Giorgi et al., 2015). A later simulation from another group using aspects of the same model investigated the impact of muscle mass and anatomy on the development of the glenohumeral joint and was able to predict the formation of brachial plexus birth injury (Dixit et al., 2020). The limitation of most simulations of joint morphogenesis is that they are based on simplified or extrapolated cell activities. Our simulations and those of others (Dixit et al., 2020; Giorgi et al., 2014; Shefelbine & Carter, 2004) have modelled the biological contribution to growth as being proportional to chondrocyte density, based on a study by Heegaard et al. (1999), in which chondrocyte density was approximated from the grey level distribution of a section of a human interphalangeal joint. As cellular processes orchestrate any changes in joint shape, the lack of a more precise and specific characterisation of cell‐level activities to joint growth and morphogenesis is a striking gap. Simulations of joint growth and morphogenesis based upon accurately tracked cell activities will provide insights into the mechanisms underlying prenatal joint growth and morphogenesis.

There is a growing body of research quantifying cellular dynamics involved in growth and morphogenesis using computational tools. Rubin et al. (2021) built 3D maps of cell morphologies from light‐sheet images of the embryonic murine tibia. Cell density, surface area, volume and orientation were quantified and spatially analysed revealing that differential cell volume expansion underlies tissue morphogenesis of the developing growth plates. Stern et al. (2022) quantified cell dynamic behaviours, such as proliferation and intercalation, in the epithelial sheet of the Drosophilia embryo and evaluated their impact on gastrulation in terms of area expansion and tissue stretching. Heller et al. (2016) developed an automated image analysis toolkit for epithelial tissues called EpiTools which enables spatial and temporal morphometric analyses of time lapse images taken at high temporal and cellular resolution—namely cell surface area, shape, division, orientation and intercalation. Applied to Drosophilia wing imaginal disc, this toolkit provided a new understanding of the role of cell rearrangements underlying tissue growth and morphogenesis. Others have been able to directly quantify tissue growth based on cell level data using lineage tracing (Marcon et al., 2011; Morishita et al., 2015; Suzuki & Morishita, 2017; Tozluoglu et al., 2019). Quantitative maps of tissue deformation coupling growth rates and anisotropy were obtained in developing chick limbs (Marcon et al., 2011; Morishita et al., 2015; Suzuki & Morishita, 2017) and in the Drosophilia wing disc (Tozluoglu et al., 2019). These studies showed that spatially and temporally heterogenous growth patterns as well as growth anisotropy are key drivers of morphogenesis, while uniform growth rates do not lead to correct shape predictions. We are not aware of any similar studies quantifying the cellular dynamics of joint morphogenesis. Such characterisation combined with computational simulations of joint growth will help us to unravel different contributions to joint morphogenesis, including the roles of cell volume changes and rearrangements as previously highlighted in other growing tissues.

In this research, we quantify the cell‐level dynamics during joint morphogenesis by tracking cell activities in high resolution in larval zebrafish jaws, then synthesise them in a predictive computational simulation of joint development. We use the simulation to test if growth heterogeneity or growth orientation is the dominant influence on joint growth and morphogenesis. This paper addresses the gap in knowledge on the cellular processes and dynamics leading to morphogenesis of developing joints.

## 2 METHODS

### 2.1 Zebrafish husbandry/zebrafish lines

Fish were maintained as described in Aleström et al. (2020). All experiments were approved by the local ethics committee (Bristol AWERB) and performed under a UK Home Office Project Licence. Transgenic lines *Tg(col2a1aBAC:mCherry)* (Mitchell et al., 2013) and *Tg(−4.9sox10:eGFP)* (Carney et al., 2006) have been previously described. These transgenic markers allow the expression of fluorescent reporters for the immature chondrocytes in the interzone (sox10‐positive and col2‐negative) and the mature chondrocytes (positive for both sox10 and col2). Both reporters are expressed in the cytoplasm and as such label the whole cell.

### 2.2 Characterising growth from cell‐level data in zebrafish jaw joints

#### 2.2.1 Zebrafish jaw joint live imaging

**FIGURE 1:**
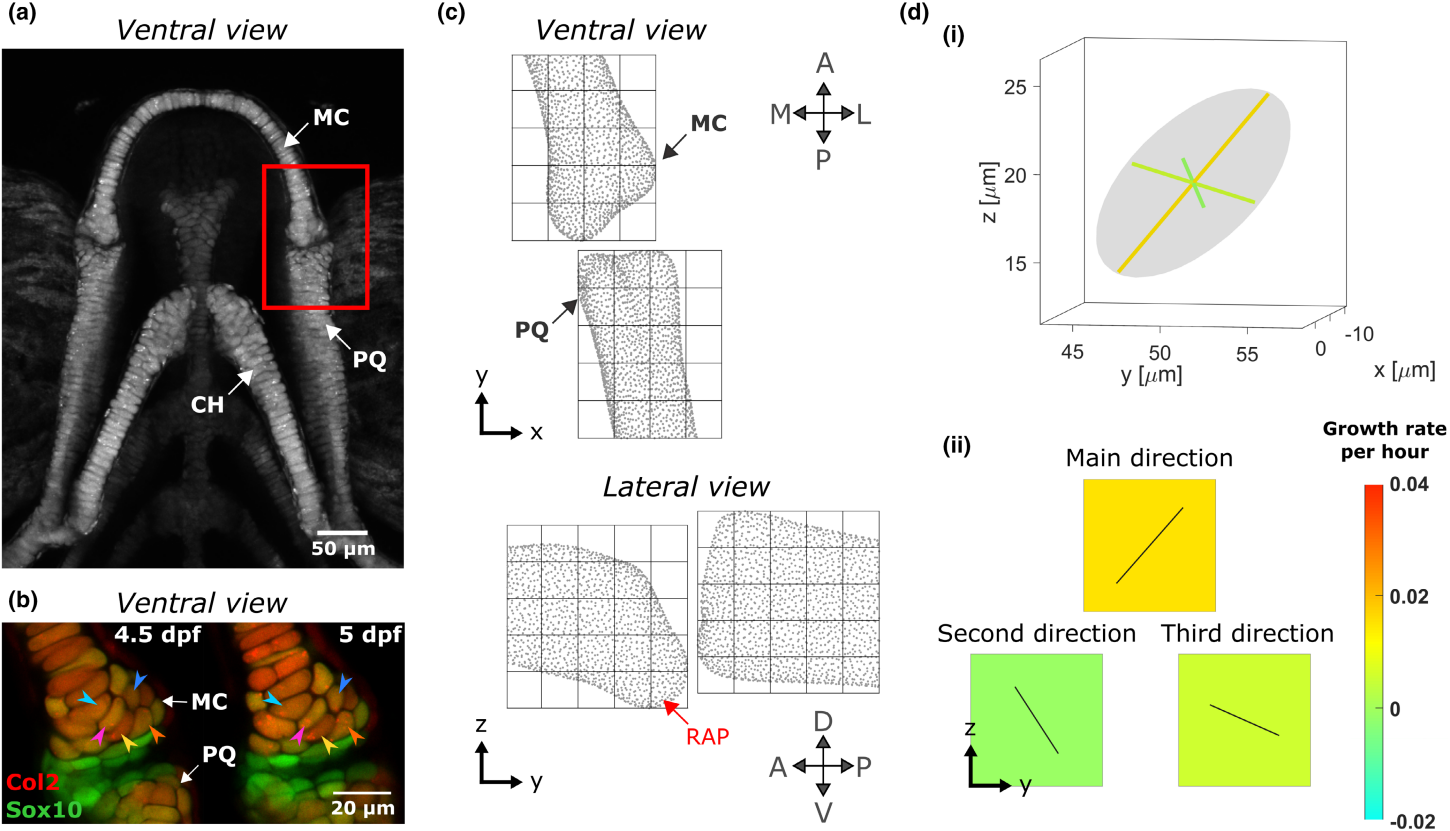
Growth map calculations in larval zebrafish jaw joint. (a) Maximum projection of ventral confocal image stacks of the jaw from a larval zebrafish aged 5 dpf expressing *Tg(Col2a1aBAC:Mcherry)* cartilage marker; red box shows the jaw joint for which morphogenesis is characterised in this study. (b) Representative ventral stacks of the anterior jaw joint element of a live specimen aged 4.5 and 5 dpf expressing the transgenic reporters *Col2a1aBAC:Mcherry* (red) and −*4.9sox10:eGFP* (green) marking cartilage in which cells can visually be identified over time. A few cells were marked by arrows as examples. Green cells are sox10+ve and col2‐ve and therefore less differentiated than the more mature yellow cells co‐expressing the two transgenes and which form the cartilaginous joint elements. (c) A grid marks out the regions (ROIs) of the anterior MC and posterior PQ joint elements in which growth is characterised. Each cube side length is 15 μm. (d) (i) The growth rate calculated for each ROI is represented by an ellipsoid with orthogonal axes. (ii) The ellipsoid's radii and the orientation of its axes are used to generate a growth map for each of the ellipsoid's radii in the lateral plane; growth rate is represented by the square's colour, while the direction of growth is shown by solid black lines in the corresponding square. A, anterior; CH, ceratohyal; D, dorsal; L, lateral; M, medial; MC, Meckel's cartilage; P, posterior; PQ, palatoquadrate; RAP, retroarticular process; V, ventral

Ten jaw joints from double transgenic *Tg(col2a1aBAC:mCherry;* −*4.9sox10:eGFP)* larvae were imaged at 12‐h intervals from 3.5 to 5.5 days post fertilisation (dpf) using a Leica SP8 confocal microscope with a temperature‐controlled chamber set to 28°C. Images centred on the joint line, as marked by a red box in Figure 1a, were acquired with a 20x HCX PL APO lens at a resolution of 512 × 512 px. Prior to imaging, larvae were anaesthetised in 0.1 mg ml^−1^ tricaine methanesulphonate (MS222) and mounted in a ventral orientation in warm 1% low melting point (LMP) agarose. Following imaging, the larvae were flushed from the agarose using Danieau's buffer, allowed to resume normal movement, and kept in separate wells of a 24‐well plate between imaging timepoints.

#### 2.2.2 Cell segmentation and tracking

Consecutive image stacks with *sox10:eGFP* chondrocyte marker were filtered in Fiji (Schindelin et al., 2012). 3D Fast Filters‐OpenGray, 3D Edge and Symmetry and 3D Morphological filters from the 3D ImageJ Suite plugin (Ollion et al., 2013) were applied in that order using the parameters supplied in Table 1. Once filtered, morphological segmentation followed by Inertia Ellipsoid filtering using Fiji's MorpholibJ plugin (Legland et al., 2016) was performed to extract the 3D cell centroids' coordinates in the joint at each timepoint. Segmentation results were then cleaned and used to manually track joint cells between images from two consecutive timepoints using manual labelling in MATLAB (R2018a, The MathWorks, Inc.). An example of how cells were visually identifiable over time is shown in Figure 1b. Cells which were col2‐negative (showing up as green in Figure 1b) were considered part of the interzone and not tracked. Due to image resolution and segmentation quality some image stacks were discarded from the analysis, and the final sample numbers per timepoint are detailed in Figure 4. At each timepoint, cells in the joint were counted to assess proliferation, and the volume occupied by the tracked cells was calculated to assess cell volume expansion.

**TABLE 1:** Filters applied to larval zebrafish jaw joint image stacks before cell segmentation

Filters	Parameters
3D fast filters‐opengray	Isotropic radius: 2 pixels
3D edge and symmetry	Canny: 0.6
3D morphological filter	Operation: closing Element shape: diamondIsotropic radius: 2 pixels

#### 2.2.3 Growth maps calculations

For each 12‐h interval time window (3.5–4, 4–4.5, 4.5–5 and 5–5.5), joint shapes were extracted from the consecutive image stacks with *col2a1:mcherry* chondrocyte marker in Mimics (Materialise NV, Leuven, Belgium) and aligned in 3‐matic (Materialise NV, Leuven, Belgium). Any transformation which was applied to the joint shapes in 3‐matic was consistently applied to the corresponding centroids in MATLAB. A cubic grid of side length 15 microns was superimposed on the aligned joints to divide them into regions of interest (ROIs) as shown in Figure 1c. For each ROI, cells within the ROI's limits were detected and their adjoining cells were listed. Vectors linking the centroids of adjacent cells were created. In each of the ROIs, a ‘statistical velocity gradient’ was calculated based on vector length and orientation variations between images from consecutive timepoints using the method described by Graner et al. (2008). This gradient quantifies local distortions and rearrangements, such that if cells within an ROI grow or intercalate, or if extracellular matrix is added, the distance between cell centroids, and therefore the geometry of the tissue, change. The statistical velocity gradient can be represented by an ellipsoid with orthogonal axes, as illustrated in Figure 1d. The orientation of the ellipsoid axes and the ellipsoid radii correspond respectively to the direction and rate of local tissue geometry deformation. Maps of local strain rates with the associated directions of deformation were generated from each of the three ellipsoid's axes as shown in Figure 1d. These maps are referred to hereafter as growth maps. Growth maps were calculated for each of the samples at each time window, and then averaged. When subject‐specific strains were used in the finite element models (see next section), the shape predictions often exhibited excessive local deformations due to noticeably higher or lower local growth rates. This provided justification for calculating averaged growth maps for each time window for use in the finite element models. Within each ROI, strain rates that lay outside the interquartile range were removed from the averaging.

Raw, filtered and segmented confocal image stacks, along with MATLAB codes for cell tracking and growth rate calculations are available at https://doi.org/10.5281/zenodo.5769854.

### 2.3 Simulating zebrafish jaw growth with a finite element model

#### 2.3.1 Zebrafish jaw joint as a model for synovial joint morphogenesis

The zebrafish jaw joint acquires a synovial‐like morphology during development (Askary et al., 2016). It has been used as a model for the study of the development of synovial joints and the aetiology of diseases such as osteoarthritis and developmental dysplasia of the hip (Lawrence et al., 2018; Roddy et al., 2017). While cavitation of the zebrafish jaw joint occurs later in development compared to in a mammalian synovial joint, the majority of morphological changes in the zebrafish jaw joint take place in the timeframe examined in this research. Therefore, the zebrafish jaw joint is a suitable model for studying the early processes of synovial joint morphogenesis.

#### 2.3.2 Shape generation

**FIGURE 2:**
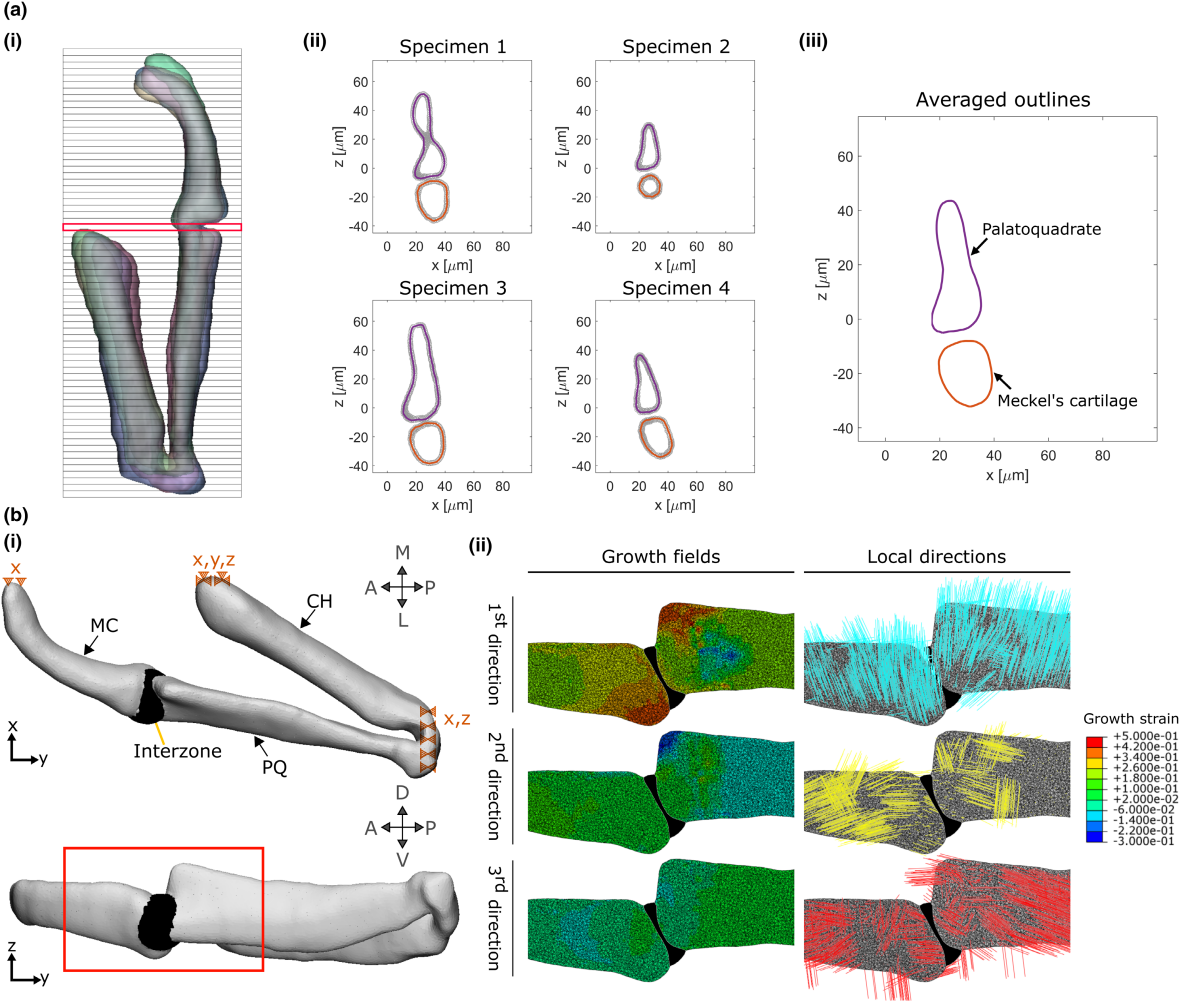
Integration of the growth maps in a finite element model. (a) The first step in constructing our FE model is to obtain an average geometry for each timepoint (3.5, 4, 4.5, 5 and 5.5 dpf). For each timepoint, half jaw shapes are aligned and sliced transversally (i). For each slice, the shape outlines of each sample (four here) are obtained (ii) then averaged (iii). The slice marked in red in (i) is shown as an example in (ii) and (iii). (b) (i) An FE model is generated based on the averaged shape outlines; the joint interzone is added and the areas marked with dashed triangles are constrained in the specified dimensions (e.g. x). (ii) Section of the joint in the lateral plane showing the growth fields which are applied to the model along with their associated directions. The view is marked by a red box in (i). A, anterior; CH, ceratohyal; L, lateral; M, medial; MC, Meckel's cartilage; P, posterior; PQ, palatoquadrate; D, dorsal; V, ventral

Confocal image stacks of four to five larval zebrafish jaws (encapsulating the Meckel's cartilage, the palatoquadrate and the ceratohyal, see Figure 1a) from the transgenic line *Tg(col2a1aBAC:mCherry)* were taken with a Leica SP8 confocal microscope at the ‘endpoints’ of each time window (3.5, 4, 4.5, 5 and 5.5 dpf) using the methodology described above. A 3D Gaussian grey filter with isotropic radius 3.0 pixels was applied to the image stacks in Fiji. These were imported into Mimics to be segmented and the resulting 3D surfaces were aligned. Only half‐jaws (separated at the level of the midsagittal plane) were segmented, as shown in Figure 2a(i). The half‐jaws were imported into MATLAB and were divided into slices in the transversal plane as shown in Figure 2a(i). For each slice, a shape outline was obtained for each specimen from the shape vertices and an average outline was generated as shown in Figure 2a(ii–iii). Averaged shape outlines were saved as image stacks and imported into Mimics where the resultant average half‐jaw shape was generated. Also in Mimics, the interzone was added as a volume filling the gap between the two joint elements using Boolean operations, with the interzone's external boundaries approximated based on imaging data (Brunt, Roddy, et al., 2016; Brunt, Skinner, et al., 2016). Finally, a non‐manifold assembly combining the half‐jaw and the interzone was generated as shown in Figure 2b(i). In 3‐matic, the non‐manifold assembly was meshed with 10 node tetrahedral elements and exported to Abaqus CAE (Dassault Systemes, 2019) where a model for each 12‐h time window was created.

#### 2.3.3 Material properties and boundary conditions

All cartilaginous regions (Meckel's cartilage (MC), palatoquadrate (PQ) and ceratohyal) were assigned homogeneous isotropic elastic material properties with Poisson's ratio 0.3 and Young's Modulus (YM) 54.8 kPa based on nanoindentation measurements taken on 5 dpf wild‐type zebrafish jaw joints (Lawrence et al., 2021). The interzone was assigned isotropic elastic material properties with Poisson's ratio 0.3 and YM set at 0.25% of the cartilaginous YM based on Brunt et al.’s original study where this ratio between the two YM was found to facilitate physiological jaw displacements when muscle loading was applied (Brunt et al., 2015). The ceratohyal does not form part of the region of interest of the jaw joint (see Figure 1), but was needed for implementing physiological boundary conditions. The following boundary conditions were applied, as illustrated in Figure 2b(i). The anterior end of the ceratohyal was fixed in all directions and translations in the lateromedial direction of the anterior ends of the Meckel's cartilage were prevented to maintain the symmetry with the missing half‐jaw. At the posterior end of the palatoquadrate, where the lower jaw connects to the rest of the craniofacial skeleton, only anteroposterior translations were allowed.

#### 2.3.4 Growth maps integration

For each 12‐h period, strains derived from the growth maps were imported into Abaqus CAE as three distinct analytical mapped fields and applied to the model. The coordinates of the ROI centres were assigned the calculated strains and interpolation was performed between ROI centres to assign strains to each element lying within the ROIs' limits. Local material orientations matching the local directions for growth were assigned to the joint elements. Elements whose nodes' coordinates were contained within an ROI were all assigned the directions for the growth of this ROI. Direction 1 is the main direction for growth (corresponding to the major axis of the statistical velocity gradient's ellipsoid), direction 2 is the second direction for growth (median axis) and direction 3 is the third direction for growth (minor axis). These directions differed from ROI to ROI. As an example, growth fields and their associated directions at the level of the joint for time window 4–4.5 dpf are shown in Figure 2b(ii). MC and PQ hypertrophic regions were not visible in the cell tracking data, but were included in the FE model of the half‐jaw. For these hypertrophic regions, growth rates were set to the average of those of a 30 μm depth of the adjacent proliferative cartilage. In the PQ hypertrophic cartilage, the material orientation of the adjacent proliferative region was used throughout. In the MC hypertrophic region, in which cell orientation varies along the length of the rudiment as shown in Figure 1a, the material orientation of the adjacent cartilage was rotated based on a linear regression of cell orientation with respect to distance from the joint line, fitted to discrete measurements taken in Fiji. The Abaqus user subroutine UEXPAN was used to apply spatially varying expansion based on the strain fields along the corresponding material orientations to provide a prediction of growth and shape for each time‐window.

#### 2.3.5 Quantification of simulation performance

**FIGURE 3:**
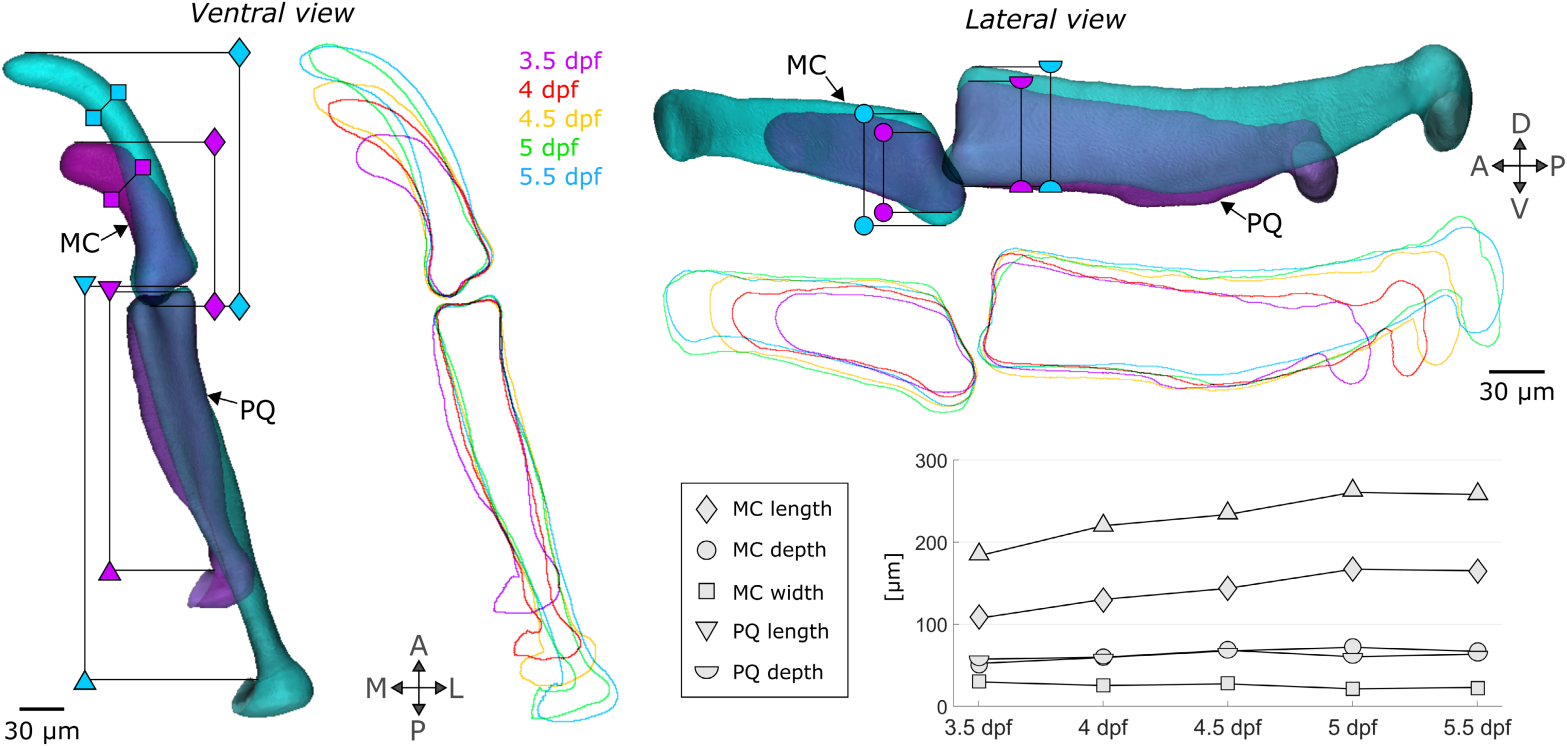
Shape changes between 3.5 and 5.5 dpf in zebrafish jaws. Superimposed 3.5 (purple) and 5.5 (turquoise) dpf 3D average shapes and 3.5 to 5.5 dpf average shape outlines in the ventral and lateral planes. The shape features which change as the jaw develops are marked with specific symbols (diamond: MC length, square: MC width, circle: MC depth, triangle: PQ length, semi‐circle: PQ depth). A, anterior; D, dorsal; L, lateral; M, medial; MC, Meckel's cartilage; P, posterior; PQ, palatoquadrate; V, ventral

The predicted shapes were imported into 3‐matic where they were aligned with the average jaw shapes of each of the ‘endpoints’ of each time window. Views in the lateral and the ventral planes were exported to Fiji where shape outlines were extracted, and the following shape features were measured: anterior Meckel's cartilage (MC) length, depth and width and posterior palatoquadrate (PQ) length and depth, as shown in Figure 3. To assess the predictive quality of the simulation for each shape feature, a percentage match of change was calculated as (a) the difference between the predicted shape measurement and the initial shape measurement divided by (b) the difference between the target shape measurement and the initial shape measurement. The following scores were then assigned based on the percentage match:

- less than 10% match: no growth predicted
- between 10% and 70%: undergrowth
- between 70% and 130%: accurate growth
- above 130%: overgrowth

#### 2.3.6 Quantification of the relative roles of growth characteristics

To quantify the relative importances of growth heterogeneity versus growth direction, simulations were conducted in which each of these features was removed or kept constant. Spatial growth heterogeneity was removed in both the MC and PQ by averaging the growth ellipsoids, within the set of ROIs in each rudiment, at each time window. In each rudiment, the average growth ellipsoid was used to generate homogeneous growth maps along the three directions for growth (corresponding to the ellipsoid's axes) and applied to the model throughout the joint and hypertrophic regions. Orientations in the MC hypertrophic region were still adapted along its length. To remove the role of orientation, isotropic growth was used. Within each ROI in both the joint and hypertrophic regions, an average growth rate corresponding to the average of the three growth ellipsoids' radii was obtained and applied to the ROI. In other words, ROIs growth ellipsoids became spheres. The resultant shapes when either growth heterogeneity or growth direction were removed were compared to the ‘full’ simulation and with each other.

MATLAB codes for shape averaging, Abaqus CAE models and real and predicted shapes are available at https://doi.org/10.5281/zenodo.5769854.

## 3 RESULTS

### 3.1 Growth in the zebrafish jaw joint exhibits spatial and temporal heterogeneity as well as marked anisotropy

**FIGURE 4:**
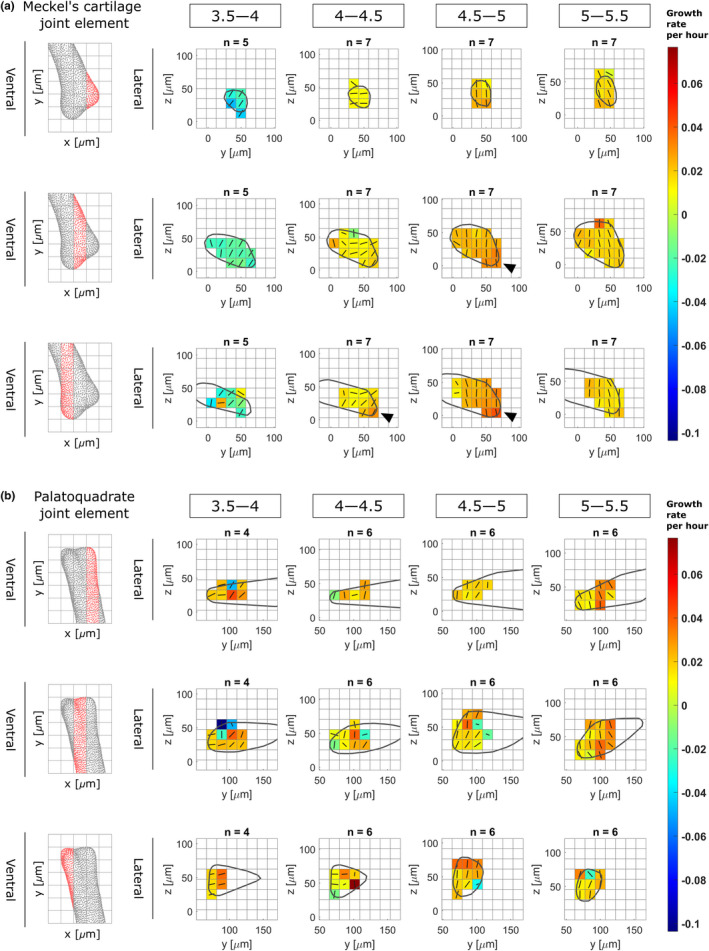
Growth rates from 3.5 to 5.5 dpf in zebrafish jaw joint exhibits spatial and temporal patterns. Maps showing growth rates along the main direction for growth (major axis of the ellipsoid) and their associated directions for each time window (3.5–4, 4–4.5, 4.5–5 and 5–5.5 dpf) in the anterior Meckel's cartilage (a) and posterior palatoquadrate (b) joint elements in the lateral plane. Growth rates are represented by colours, while the direction is shown by solid black lines. Results are displayed across the rudiment's width; views in the ventral plane of each section are displayed on the left panels. Black arrowheads show areas of elevated growth rates

Comparing shape feature measurements between 3.5 and 5.5 dpf revealed an overall volume expansion over time with a marked increase in Meckel's cartilage (MC) and palatoquadrate (PQ) length (Figure 3: diamond and triangle), a slight increase in MC and PQ depth (Figure 3: circle and semi‐circle) and a slight contraction of MC width (Figure 3: square). In the anterior MC joint element, growth rates in the main direction for growth varied between time windows, ranging from contraction at a mean rate of −2.06 ± 1.49 × 10^−2^ per hour from 3.5 to 4 dpf, to expansion at a rate of 2.45 ± 0.61 × 10^−2^ per hour from 4.5 to 5 dpf, as shown in Table 2. In the posterior PQ joint element, growth rates in the main direction consistently increased from a mean rate of 1.01 ± 3.51 × 10^−2^ per hour from 3.5 to 4 dpf to a mean rate of 2.10 ± 1.27 × 10^−2^ per hour from 5 to 5.5 dpf, as shown in Table 2. Higher variability was observed from 3.5 to 4 dpf than other time windows due to the experimental challenges of imaging the youngest zebrafish larval jaw joints and a degree of natural variation in the onset and number of jaw movements (and therefore mechanical stimuli) at earlier stages. Elevated growth rates in the main direction were observed at the retroarticular process (the most ventroposterior area of the anterior MC joint element shown in Figure 1c) from 4–4.5 to 4.5–5 dpf as shown in Figure 4a (black arrows). Growth rates along the second and third directions for growth were much lower than those of the main direction in both the MC and PQ, as shown in Table 2, demonstrating growth anisotropy. Growth maps in the second and third directions for growth are provided in Figures S1 and S2. Growth orientations in the anterior MC element exhibited consistent alignment across ROIs; with time, the main direction shifted to align with the ventrodorsal axis from 4.5–5 to 5–5.5 dpf, as shown with solid black lines in Figure 4a. The main direction for growth in the posterior PQ element also tended to align with the ventrodorsal axis from 4.5–5 to 5–5.5 dpf as shown with the black lines in Figure 4b. Overall, growth rates and orientations in the developing jaw joint changed over the time period studied in both joint elements and elevated growth rates were observed at the retroarticular process of the MC demonstrating spatial and temporal growth heterogeneity. Marked growth anisotropy was observed in both joint elements.

**TABLE 2:** Mean growth rates per hour (×10^−2^) along the three orthogonal directions for growth for each time window (days post fertilisation (dpf) 3.5–4, 4–4.5, 4.5–5 and 5–5.5) in the anterior Meckel's cartilage (MC) and posterior palatoquadrate (PQ) joint elements

	3.5–4 dpf	4–4.5 dpf	4.5–5 dpf	5–5.5 dpf
Anterior MC joint element				
Main direction	−2.06 ± 1.49	1.38 ± 0.67	2.45 ± 0.61	1.83 ± 0.48
Second direction	1.20 ± 1.07	0.39 ± 0.52	0.50 ± 0.37	0.26 ± 0.45
Third direction	0.44 ± 0.47	0.031 ± 0.24	0.09 ± 0.29	0.10 ± 0.17
Posterior PQ joint element				
Main direction	1.01 ± 3.51	1.53 ± 2.16	1.62 ± 1.62	2.10 ± 1.27
Second direction	0.54 ± 0.68	0.26 ± 0.60	0.42 ± 0.93	−0.46 ± 0.94
Third direction	0.06 ± 2.23	−0.07 ± 1.41	0.09 ± 0.43	0.07 ± 0.26

**FIGURE 5:**
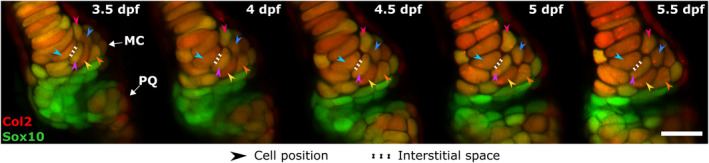
Cell intercalation and the extracellular matrix do not majorly contribute to jaw joint shaping. Representative ventral stacks of the anterior jaw joint element of a live specimen aged 3.5, 4, 4.5, 5 and 5.5 dpf expressing the transgenic reporters *Col2a1aBAC:Mcherry* (red) and −*4.9sox10:eGFP* (green) marking cartilage. Green cells are sox10+ve and col2‐ve and therefore less differentiated than the more mature yellow cells co‐expressing the two transgenes and which form the cartilaginous joint elements. No observation of cell intercalation is made with the cells being clearly identified over time (six cells marked by arrows as examples). The volume occupied by the interstitial space is minor compared to the volume occupied by cells. Scale bar: 20 μm. MC, Meckel's cartilage; PQ, Palatoquadrate

Manual assessment of tracked cells over the time window studied revealed very low proliferation rates in the joint. The percentage of cells which underwent division in the joint over 12 h was 2.42% ± 1.73% in the MC and 0.50 ± 0.56% in the PQ, suggesting that proliferation would only minorly impact on joint growth. No intercalation of joint cells was observed over the 12‐h timeframes during cell tracking (sample cell tracking over time shown in Figure 5). The volume occupied by tracked joint cells over the timeframe of interest increased substantially, with a mean relative volume expansion per 12‐h period of 18.49% ± 20.44% in the MC and 23.68% ± 23.92% in the PQ. Because the ECM forms a thin layer between adjacent cells (see Figure 5), it could not be accurately segmented and its volume was not directly quantified. However, the interstitial space between adjacent cells was consistently narrow, with no apparent increase over time (see example in Figure 5). Therefore, our data indicate that increases in joint volume over the studied time window were primarily due to cell volume increases, rather than proliferation or increases in ECM volume.

### 3.2 Cell positional information over time enables consistent prediction of zebrafish jaw morphogenesis

**FIGURE 6:**
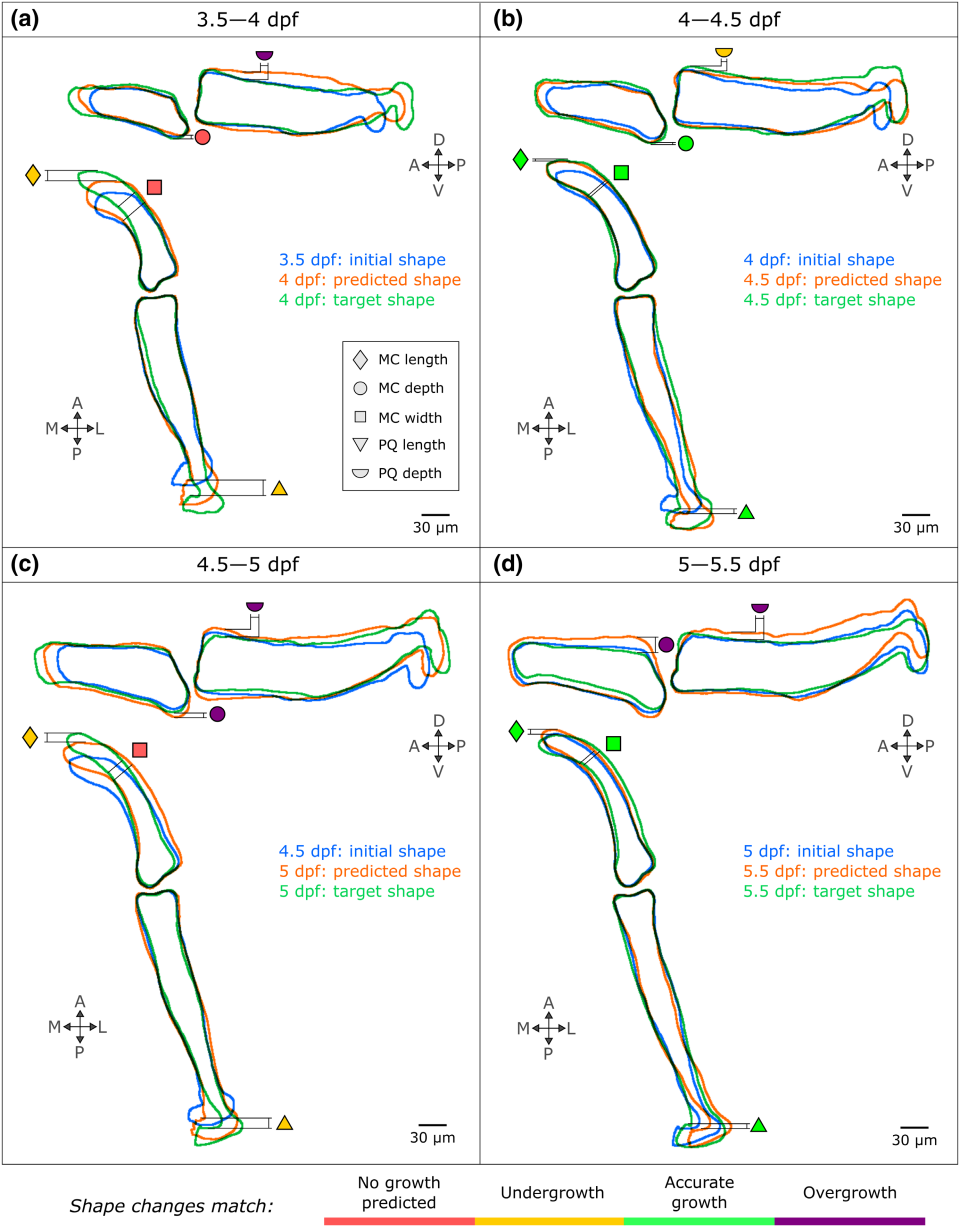
The integration of cell‐based data in an FE model successfully predicts zebrafish jaw shape changes from 3.5 to 5.5 dpf, with most faithful predictions from 4 to 4.5 dpf. The shape outlines for each time window are superimposed (blue: Initial shape, green: Target shape, orange: Predicted shape) and the shapes features introduced in Figure 3 are compared (triangle: Palatoquadrate (PQ) length, diamond: Meckel's cartilage (MC) length, square: MC width, semi‐circle: PQ depth, circle: MC depth) and rated with a colour code explained in the bottom panel (red means no growth predicted, yellow means undergrowth though the pattern of change is correct, green means accurate shape changes and violet means overgrowth though the pattern of change is correct). A, anterior; D, dorsal; L, lateral; M, medial; P, posterior; V, ventral

Growth for each of the time windows was computationally simulated based on the calculated growth maps, and the shape features undergoing change between 3.5 and 5.5 dpf were used to assess the quality of the shape predictions. For each time window, most observed shape changes were predicted, either accurately, or with some under‐ or over‐growth, as highlighted with green, yellow and purple (respectively) symbols in Figure 6. Length change in both rudiments was accurate from 4–4.5 to 5–5.5 dpf (green triangles and diamonds in Figure 6b,d) but undergrowth was observed from 3.5–4 to 4.5–5 dpf (yellow triangle and diamond in Figure 6a,c). The change of depth in the lateral plane in both rudiments was mostly predicted (yellow and purple circles and semi‐circles in Figure 6a,c,d) though only the 4–4.5 predictions accurately matched the target shape (green circle in Figure 6b). The decrease of MC width observed from 3.5–4 to 4.5–5 dpf in the ventral plane was not replicated in the predicted shapes (red squares, Figure 6a,c). Overall, the shape predictions were close to their target shapes (Figure 6), with most successful predictions from 4 to 4.5 dpf, in which all but one shape feature was scored with a green symbol (Figure 6b). The least successful predictions occurred from 3.5 to 4 dpf. In the 3.5–4 dpf timeframe, immature cells in the MC anterior tip rearrange and undergo morphological changes contributing to MC elongation and reduction in width, as shown in Figure S3. Cell arrangement changes at the anterior tip were not quantified in our study (due to the primary focus on the synovial jaw joint), which may have contributed to less accurate overall shape predictions. In conclusion, zebrafish jaw joint growth and shape change for the time window modelled can be reasonably approximated based on cell positional information over time, where that cell positional information derives mainly from cell rearrangements and volume expansion.

### 3.3 Growth orientation is more important for zebrafish jaw joint shaping than growth heterogeneity

**FIGURE 7:**
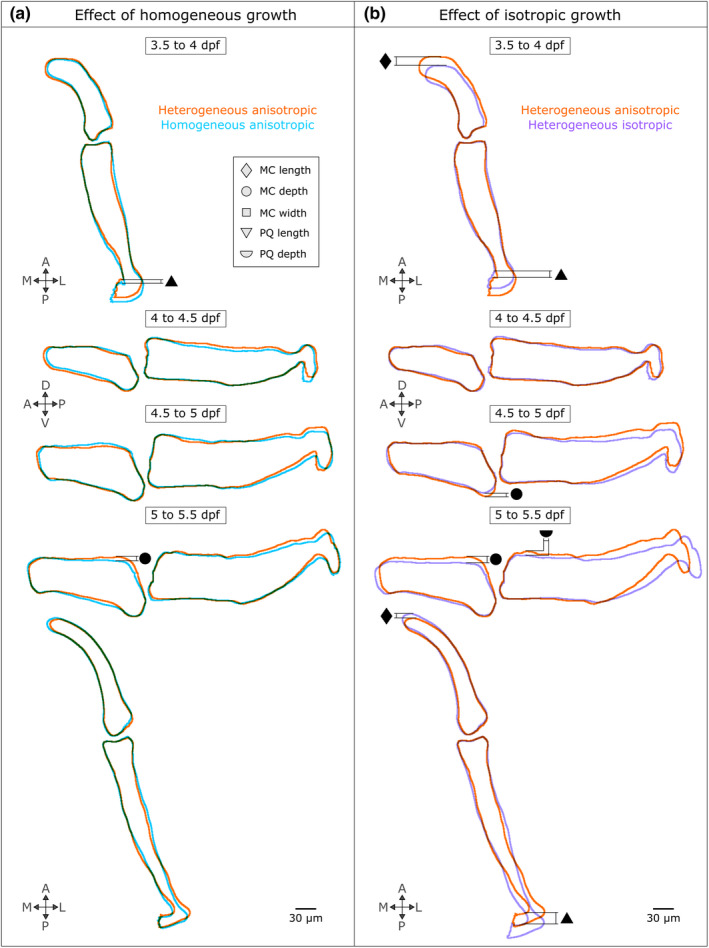
Growth orientation plays an important role in jaw joint shaping, whereas growth heterogeneity minorly impacts zebrafish jaw shape predictions. Growth predictions obtained from homogeneous anisotropic (a) and heterogeneous isotropic (b) growth fields are compared with the ‘full’ simulation (heterogeneous anisotropic). Only the views where shape changes were observed in either the homogeneous anisotropic or the heterogeneous isotropic shape predictions compared to the full simulation are displayed. The shape outlines in all views and time windows are displayed in Figures S4 and S5. The black symbols denote the shape features which have been altered when either growth heterogeneity or orientation have been removed (triangle: Palatoquadrate (PQ) length, diamond: Meckel's cartilage (MC) length, square: MC width, semi‐circle: PQ depth, circle: MC depth)

The importance of growth heterogeneity and direction was assessed in simulations in which each of these features was either removed or kept constant. Removing growth heterogeneity resulted in only minor shape changes: over the four time‐windows, two features exhibited undergrowth compared to the ‘full’ simulations (PQ length from 3.5 to 4 dpf and MC depth from 5 to 5.5 dpf as shown in Figure 7a). In contrast, when growth orientation was removed, several shape features were markedly altered compared to the ‘full’ simulation. From 3.5 to 4 dpf under isotropic growth, both MC and PQ length exhibited marked undergrowth as seen in Figure 7b. No changes due to isotropic growth were observed from 4 to 4.5 dpf, while MC depth slightly undergrew from 4.5 to 5 dpf as shown in Figure 7b. From 5 to 5.5 dpf under isotropic growth, both MC and PQ length and depth were markedly undergrown as shown in Figure 7b. The time windows most severely impacted by the removal of growth orientation (from 3.5–4 to 5–5.5 dpf) were also the windows that exhibited the most complex growth patterns with pronounced growth anisotropy (see Table 2 and Figure 4). Growth predictions for the four time‐windows and both adjusted simulation types are provided in Figures S4 and S5. These results indicate that growth orientation, and the cellular dynamics likely responsible for it, such as cell orientation and oriented cell division, are crucial to normal morphogenesis. Taken together, our findings suggest that whereas cell proliferation, intercalation and ECM deposition minorly impacted zebrafish jaw joint growth, cell volume expansion and orientation dominate joint growth and morphogenesis.

## 4 DISCUSSION

In this research, local tissue deformations of larval zebrafish jaw joints were quantified based upon tracked cell‐level data and simulated in a predictive model of joint growth. Our model, the first to simulate joint growth based on biofidelic data, was used to unravel dominant influences and identify which cellular behaviours dominate growth and morphogenesis in the developing zebrafish jaw joint.

Our analysis of zebrafish jaw joint cell dynamics revealed spatially and temporally heterogeneous growth patterns. Growth rates and orientations evolved over the time period studied and elevated growth rates were evident at the retroarticular process of the Meckel's cartilage, which is known to project ventro‐posteriorly from the jaw joint during larval development (Eames et al., 2013). In other developing tissues, such as the developing chick limb bud (Morishita et al., 2015; Suzuki & Morishita, 2017) and the drosophilia wing disc (Tozluoglu et al., 2019), spatial and temporal growth heterogeneity was shown to be a key driver of morphogenesis, and in simulations, uniform growth rates did not lead to correct shape predictions (Tozluoglu et al., 2019). In contrast to the limb bud and wing disc, our data indicate that spatial growth heterogeneity is not a dominant influence on zebrafish jaw joint shape for the time windows investigated. Rather, growth orientation was more important for jaw joint growth and morphogenesis in the timeframe studied. Our analysis of zebrafish jaw joint cell dynamics revealed a marked growth anisotropy for the time period studied, and in simulations, isotropic growth led to pronounced shape alterations. This observation is in line with Boehm et al.’s work (2010) in which a parameter optimisation approach on murine limb bud development revealed that growth orientation was critical for accurate shape prediction. Altered cell orientation and increased cell sphericity has been shown to be correlated with altered zebrafish jaw shapes which could indicate a link between cell orientation and growth orientation (Brunt et al., 2015; Brunt, Roddy, et al., 2016; Brunt, Skinner, et al., 2016; Lawrence et al., 2018).

Our quantification of cell dynamics was derived from cell rearrangements, cell volume expansion and extracellular matrix (ECM) deposition, demonstrating that joint growth and morphogenesis can be reasonably approximated based on these behaviours. Analysis of cell numbers indicated that proliferation is unlikely to be a dominant influence in the joint over the timeframe examined, despite the fact that proliferation has been highlighted in the more mature regions of the developing cartilage elements and in the interzone (Brunt et al., 2017; Kimmel et al., 1998). We also propose that cell intercalation is not likely to have a very strong influence on jaw joint growth in the timeframe and region examined, while acknowledging that cell stacking and convergent extension are key features of more mature regions of the developing cartilage elements (Brunt, Roddy, et al., 2016; Brunt, Skinner, et al., 2016; Eames et al., 2013; Kimmel et al., 1998; Mork & Crump, 2015; Shwartz et al., 2012). As previously reported (Brunt et al., 2017; Brunt, Roddy, et al., 2016; Brunt, Skinner, et al., 2016; Kimmel et al., 1998), we found that cell volume expansion is likely a key contributor to joint growth, while we found no evidence of substantial increases in ECM volume over the timeframe under investigation. This corroborates the findings of a recent study conducted on the juvenile zebrafish pharyngeal skeleton where ECM volume increase was found to be negligible (Heubel et al., 2021). The findings of this research apply to the zebrafish synovial jaw joint at the early stages of morphogenesis. Unlike mammalian synovial joints, cavitation of the zebrafish jaw joint occurs after the majority of morphological changes have taken place (Askary et al., 2016). Because of this difference, confirming the findings in the synovial joints of mammalian model systems would be valuable for generalisation.

Some failures in shape predictions were observed in our results. Cell contraction in the hypertrophic regions of the Meckel's cartilage has not been accurately simulated due to the specific cell arrangements; in the Meckel's cartilage, cells stack into a single column in the antero‐posterior axis. Because the algorithm for growth quantification does not directly account for cell shape, a medio‐lateral contraction of cells in such a columnar arrangement cannot be captured. Another imprecision in our models arose from the lack of quantification of cell morphological changes and rearrangements at the MC anterior tip which occur from 3.5 to 4 dpf and which contribute to the rudiment's growth. In some other timeframes, predicted under‐ or over‐growth of the Meckel's cartilage and the palatoquadrate length and depth resulted from the small number of cells in the zebrafish jaw. Some shape prediction imprecisions observed may also be a consequence of using average shape and strain data. If it had been feasible in terms of the methodology, predictions using subject‐specific data could have helped increase accuracy. The use of a model using a discrete number of cells, such as vertex models in which each cell is represented by a polygon (Alt et al., 2017), would have likely increased our control of the impact of each individual cell shape over time. A major application of such models is the study of epithelial morphogenesis, notably in the Drosophilia wing (Fletcher et al., 2017; Ioannou et al., 2020; Rauzi et al., 2008). An advantage of our modelling approach compared to cell‐based models is the capacity to apply it to organisms with much greater numbers of cells. Because our model focusses on macro‐scale shape changes and does not simulate individual cell behaviours, it ensures computational simplicity and practicability, whereas cell‐based models are constrained to a limited number of cells.

Our method as presented here is optimal for specimens in which live imaging can be performed. A straightforward application is to quantify growth patterns in epithelial tissues using high cellular resolution images obtained through fluorescence microscopy combined with automated tools for cell segmentation and tracking like EpiTools (Heller et al., 2016). Modelling axolotl joint growth using our approach is also feasible. The axolotl is often used as a model for limb development (Hutchison et al., 2007; Nye et al., 2003) and progress has been made in visualising cells at high resolution during live imaging (Masselink & Tanaka, 2021). The existence of rainbow transgenic lines also facilitates cell tracking and visualisation and was used in the past to study digit tip regeneration (Currie et al., 2016). Although live imaging is optimal, it may not be critical to track individual cells with larger numbers of cells. Comparisons between local tissue geometry at successive timepoints may be sufficient to predict joint growth and morphogenesis, which we are exploring in ongoing work.

In conclusion, our findings show that cell volume expansion and orientation are key drivers of larval zebrafish jaw joint growth and morphogenesis. These new insights on what drives joint growth and morphogenesis were facilitated through growth predictions based upon precise and specific cell‐level characterisation of growth. Gaining a better understanding of the cell‐level processes and dynamics of joint morphogenesis opens up new avenues towards understanding the aetiology of congenital conditions such as developmental dysplasia of the hip and arthrogryposis.

## AUTHORS’ CONTRIBUTIONS

J.G., C.L.H. and N.C.N. carried out conceptualisation, analysis and visualisation. J.G., E.A.L., M.W., C.L.H. and N.C.N. were involved in methodology and writing—reviewing and editing of the manuscript. J.G. also carried out software design and implementation. C.L.H. and N.C.N. also carried out supervision and funding acquisition of the manuscript. J.G. and N.C.N. also contributed to writing—original draft of the manuscript.

### OPEN RESEARCH BADGES

This article has earned Open Data and Open Materials badges. Data and materials are available at https://doi.org/10.5281/zenodo.5769854.

## Supporting information


Figure S1
Figure S2
Figure S3
Figure S4
Figure S5

## Data Availability

Data underlying this article can be accessed on zenodo at https://doi.org/10.5281/zenodo.5769854.
